# Pharmacokinetics study of atracurium, dexmedetomidine, midazolam and 1-hydroxymidazolam in patients undergoing acute aortic dissection surgery

**DOI:** 10.3389/fphar.2024.1427553

**Published:** 2024-10-24

**Authors:** Huiling Si, Xuanxuan Xu, Yuhao Liang, Shuaibo Shi, Fan Xie, Jie Hu

**Affiliations:** ^1^ Department of Anesthesiology, Luoyang Central Hospital Affiliated to Xinxiang Medical University, luoyang, China; ^2^ Department of Anesthesiology, The Second People’s Hospital of Panyu, Guangzhou, China; ^3^ Department of Anesthesiology, Luoyang Central Hospital, Luoyang, China

**Keywords:** dexmedetomidine, atracurium, midazolam, 1-hydroxymidazolam, UPLC-MS/MS, pharmacokinetics

## Abstract

**Objective:**

An UPLC-MS/MS method was developed and validated for simultaneous determination of atracurium (ATC), dexmedetomidine (DEX), midazolam (MDZ) and 1-hydroxymidazolam (1-OH-MDZ) and the pharmacokinetics of ATC, DEX, MDZ and 1-OH-MDZ in patients undergoing aortic dissection surgery were investigated.

**Methods:**

The analytes were extracted by acetonitrile precipitation and separated on an Acquity UPLC BEH C18 column (2.1 mm × 50 mm, 1.7 μm) with a mobile phase of acetonitrile-0.1% formic acid and a gradient mode. In the positive ion mode, the following mass transition pairs were monitored by multiple reaction monitoring (MRM) for the four analytes and IS: m/z 385.1→206.2 for ATC, m/z 201.2→95.1 for DEX, m/z 326.1→291.1 for MDZ, m/z 341.9→324.0 for 1-OH-MDZ, and 284.9→153.9 for diazepam (IS). Seven male patients undergoing aortic dissection surgery received general anesthesia and intravenous administration of ATC, DEX, and MDZ during the surgery. Venous blood was collected at different time points at the end of surgery and after surgery. The concentrations of ATC, DEX, MDZ, and 1-OH-MDZ were detected, and the pharmacokinetic parameters were calculated.

**Results:**

The method showed good linearity for each analyte. The inter-batch precision ranged from 1.37% to 9.87% and the intra-batch precision ranged from 2.41% to 10.72%; the accuracy ranged from 94.33% to 104.51%. Finally, the matrix effect, extraction recovery and stability data met the FDA recommended acceptance criteria for validation of bioanalytical methods. The t_1/2_ of ATC, DEX, MDZ and 1-OH-MDZ was (6.74 ± 2.27) h, (9.55 ± 4.93) h, (10.17 ± 5.35) h, and (6.90 ± 2.38) h, the C_max_, of ATC, DEX, MDZ and 1-OH-MDZ was (1054.20 ± 202.37) ng/mL, (1.93 ± 1.07) ng/mL, (1256.57 ± 389.09) ng/mL, and (1034.39 ± 292.92) ng/mL in patients undergoing aortic dissection surgery, respectively.

**Conclusion:**

The developed UPLC-MS/MS method for simultaneous determination of ATC, DEX, MDZ and 1-OH-MDZ in patient plasma was accurate, reproducible, specific. After continuous administration of ATC, DEX, and MDZ to patients undergoing surgery for acute aortic dissection, the pharmacokinetics of ATC, DEX, MDZ and 1-OH-MDZ in patients undergoing aortic dissection surgery were studied.

## 1 Introduction

Acute aortic dissection (AAD) has a poor prognosis, and requires early diagnosis, appropriate treatment strategies, and detection of symptoms with suspicion; As such, this condition is often described as an acute aortic emergency (Tao et al., 2023). AAD is a highly fatal cardiovascular emergency, defined as the gradual separation of the aortic layer due to interstitial degeneration of the aorta (Rylski et al., 2023). AAD has a significant impact on mortality and incidence rate (Wai Sang et al., 2020). The treatment of AAD is stratified based on the location of the lesion: Type A requires surgical intervention, while Type B, despite its complexity, can accept medication treatment (Erbel et al., 2014). Performing emergency surgical evaluation and repair is required for AAD. Intraoperative management of AAD requires the anesthesiologist to do more than just anesthesia, but begins before the patient arrives in the operating room. High-fidelity communication with the surgeon, knowledge of surgical planning, the anatomy of aortic coarctation, and a subtle understanding of the pathophysiology of aortic coarctation are all critical aspects of anesthesia management (Stombaugh and Mangunta, 2022). Anesthetics and anesthesia adjuncts are also important aspects of surgical anesthesia management. In clinical practice, adjuvants are widely used to prolong anesthesia/analgesia, stabilize hemodynamics, reduce postoperative pain, and decrease postoperative complications (Chen Z. P. et al., 2023). Atracurium, dexmedetomidine, and midazolam are commonly used anesthesia adjuncts during surgery.

**FIGURE 1:**
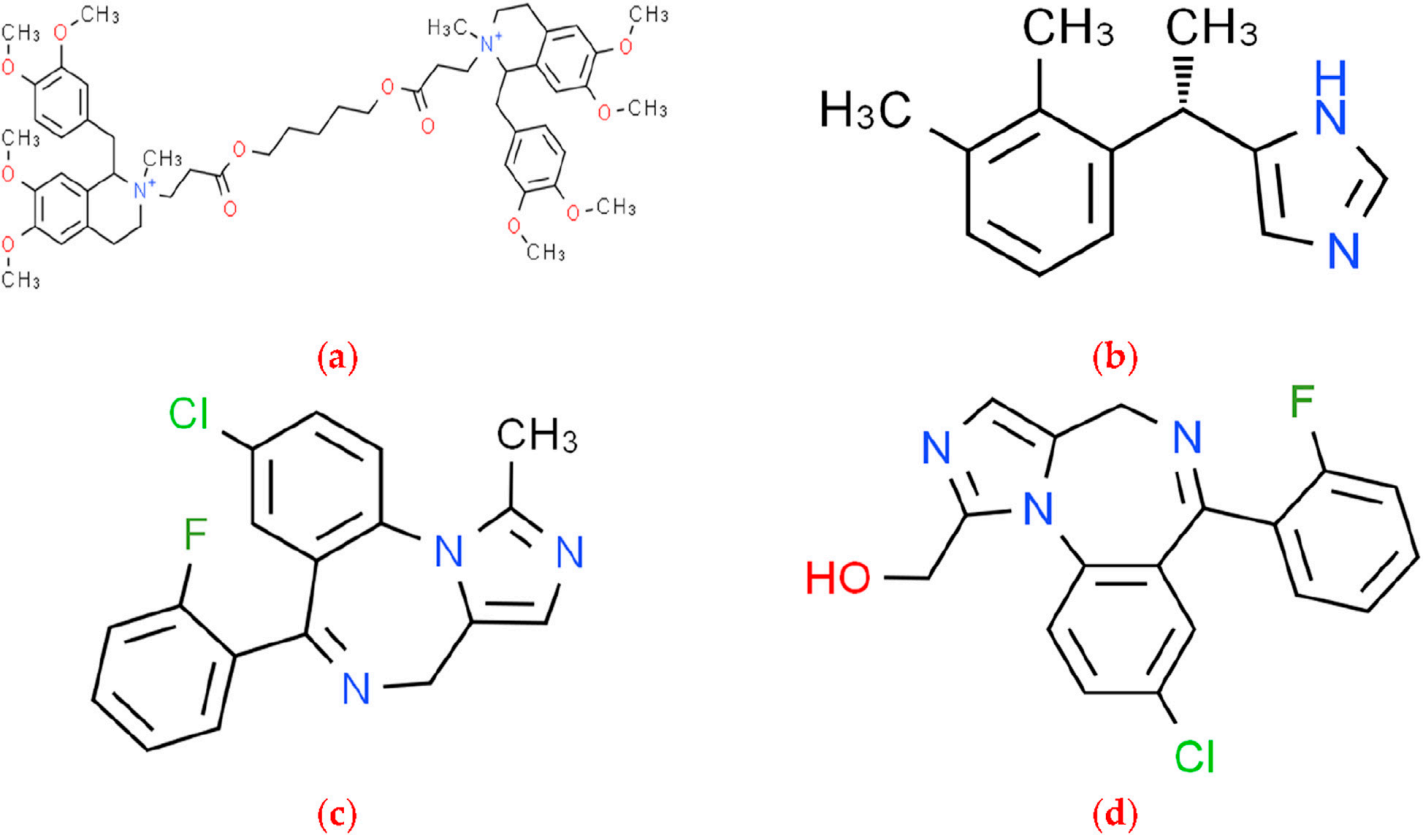
The chemical structure of four analytes: **(A)** ATC; **(B)** DEX; **(C)** MDZ; **(D)** 1-OH-MDZ.

Atracurium (ATC, Figure 1A) is a non depolarizing neuromuscular blocker of the benzylisoquinoline class. ATC is considered a supplement to general anesthesia, facilitating tracheal intubation and providing skeletal muscle relaxation during surgery or mechanical ventilation (Ritz et al., 2023). The use of high-dose ATC (1 mg/kg) during rapid sequence induction can achieve acceptable intubation conditions within 1 min. ATC can serve as an alternative drug for rapid sequence induction (Holkunde et al., 2022). In some cases, administering relatively high doses of ATC without triggering can serve as an alternative to neuromuscular blockers for rapid sequential induction of anesthesia (Chalermkitpanit et al., 2020). At a dose of 0.4 mg/kg of ATC, the success rate of the first attempt to insert the laryngeal mask airway was high, accompanied by lower respiratory complications (bleeding and sore throat). Increasing this dose has no significant effect on the success rate of laryngeal mask airway implantation (Shetabi et al., 2020). Meanwhile, in the treatment of severe acute respiratory distress syndrome, ATC seems to be a safe and inexpensive alternative drug (Carabetta et al., 2023).

Dexmedetomidine (DEX, Figure 1B) is a selective α2-adrenoreceptor agonist with a broad range of effects, including easily controllable sedation, analgesia and anxiolysis. Due to these advantageous characteristics, traditional sedatives such as benzodiazepines have been replaced by DEX as the first line of sedation for intensive care unit patients (Mano et al., 2023). As an adjuvant drug, DEX offers significant advantages in brachial plexus block, paravertebral block and transversus abdominis block, which are characterized by prolonged analgesic time (Chen Z. P. et al., 2023). The administration of intraoperative and postoperative DEX reduced the incidence of post-traumatic stress disorder (PTSD) among patients with trauma. The findings of this trial support the use of DEX in emergency trauma surgery (Yu et al., 2023). The use of DEX as a cardioprotective drug can further promote the management of patients undergoing cardiac surgery and can be considered in a broader clinical environment beyond cardiac surgery, including patients with acute myocardial infarction (Takahashi et al., 2023). DEX pretreatment can protect against cardiac ischemia reperfusion injury, presumably by promoting STAT3 phosphorylation via the α2-adrenoreceptor *in vivo* and *in vitro* (Chen Z. R. et al., 2023). DEX inhibits iron removal through Nrf2/GPX4 pathway, alleviates high glucose induced myocardial cell injury, and provides potential therapeutic targets for the treatment of diabetes cardiomyopathy (Li et al., 2023). Anesthesia induced neurotoxicity is a set of adverse side effects related to anesthesia administration on the central or peripheral nervous system. DEX has a unique position in avoiding potential neurotoxicity associated with anesthesia (Tsivitis et al., 2023).

Midazolam (MDZ, Figure 1C) is an intravenous benzodiazepine drug used as a sedative anesthetic for minor surgeries and an adjunct to general anesthesia (LiverTox, 2023). Intravenous MDZ is used for induction of anesthesia and for control of acute seizures. Because of water-soluble, MDZ has a rapid onset of action and can be used to control status epilepticus when other drugs cannot be administered intravenously. During the maintenance phase of general anesthesia, MDZ can be used as an anxiolytic and hypnotic. MDZ is an adjunct to regional and local anesthesia and can be used for a variety of diagnostic and therapeutic procedures with greater patient and clinician acceptance (Lingamchetty et al., 2023). MDZ is a well-established substrate of CYP3A4 and is often used both *in vitro* and *in vivo* as a probe for potent inhibitors of CYP450. The main product of MDZ metabolism, 1-hydroxymidazolam (1-OH-MDZ, Figure 1D), is commonly used as a probe for competitive binding of various drugs to CYP3A4 (Denisov et al., 2022; Denisov et al., 2021).

Anesthetic techniques and agents used during surgery to accelerate weaning from mechanical lung ventilation and patient recovery are essential for rapid cardiac anesthesia and are being increasingly adopted. ATC, DEX, MDZ are all anesthetic adjuvant drugs, which may be used simultaneously in clinical practice, but there is currently no method for simultaneously detecting the concentrations of these drugs. In the current research, a fast and sensitive UPLC-MS/MS method for the simultaneous determination of ATC, DEX, MDZ and 1-OH-MDZ was developed, and the pharmacokinetic profiles of ATC, DEX, MDZ, and 1-OH MDZ in patients undergoing AAD surgery were described in our study.

## 2 Materials and methods

### 2.1 Chemicals and reagents

ATC (purity > 98.0%), DEX (purity > 98.0%), MDZ (purity > 98.0%), 1-OH-MDZ (purity > 98.0%) and were purchased from Sigma (St.Louis, MO, United States). Diazepam (purity > 98.0%, internal standard, IS) were obtained from China Academy of pharmaceutical and biological products. Methanol and acetonitrile of LC-grade were provided by Tianjin Kermel Chemical Reagent Co., Ltd. Formic acid were procured from Sigma-Aidrich.

ATC Injection (LOT NO: 22042731) was purchased from Jiangsu Hengrui Pharmaceutical Co., Ltd. DEX Injection (LOT NO: 220222BP) was obtained from Jiangsu Hengrui Pharmaceutical Co., Ltd. MDZ Injection (LOT NO: 22180311) was purchased from Yichang Renfu Pharmaceutical Co., Ltd.

### 2.2 Instruments

UPLC instrument was Waters ACQUITY UPLC instrument including a chromatographic column manager, a column temperature box with heating and cooling functions, a binary solvent manager, and a sample manager. Mass spectrometric (MS) instrument was Waters Xevo TQ-S triple quadrupole mass spectrometer with electric spray ion (ESI) source.

### 2.3 General information of patients

The experimental plan had been approved by the Ethics Committee of Luoyang Central Hospital, Henan Province. Seven patients who underwent surgery for aortic dissection were all from the Department of Cardiology, Luoyang Central Hospital, Henan Province. The patients provided written informed consent to participate in this study. The 7 patients were all male, aged 38–71 years old, with the height of 158–172 cm and the weight of 58–80 kg. The 7 patients had normal blood and urine routine tests, as well as normal liver and kidney function tests.

The anesthesia method was general anesthesia, and the first surgical name was coronary artery bypass grafting. Before surgery, 10 mg of morphine and 0.3 mg of scopolamine were administered. The general information of the patient was shown in Table 1.

**TABLE 1:** The general information of the patients.

Number	Gender	Age (y)	Height (cm)	Weight (kg)	Body mass index	Preoperative BP (mmHg)	Preoperative HR (Times/min)
1	Male	71	168	80	28.35	141/84	54
2	Male	69	169	72	25.21	127/74	68
3	Male	63	170	63	21.80	125/79	90
4	Male	70	167	60	21.51	109/71	80
5	Male	71	170	60	20.76	150/84	48
6	Male	59	158	58	23.23	100/60	68
7	Male	38	172	70	23.66	101/67	82

### 2.4 Medication and blood collection

After pretreatment with intramuscular injections of 10 mg morphine and 0.3 mg scopolamine, patients were anesthetized with etomidate (0.3 mg/kg), sufentanil (0.8 μg/kg), and ATC (0.2 mg/kg), as well as endotracheal intubation and mechanical ventilation of the lungs (I/E = 1:1.5; VT = 6–8 mL/kg; 10–12/min). During anesthesia, DEX was prepared at a concentration of 4 μg/mL and slowly infused intravenously at a dose of 1 μg/kg/hr for 10 min, and then reduced to maintenance dose of 0.3 μg/kg/hr. All patients also received MDZ (0.1 mg/kg) and ATC (0.1 mg/kg), if necessary, for the duration of the procedure. Patients were routinely monitored, including invasive arterial pressure, pulmonary artery pressure, cardiac index, and cardiac output were continuously monitored through a Swan-Ganz catheter (Vigileo II, Edwards, Irvine, United States).

2 mL of venous blood was collected from each patient at the end of surgery and 0.17, 0.5, 1.0, 1.5, 2.0, 3.0, 4.0, 6.0, 9.0, 12.0, and 24.0 h after surgery. After centrifugation at 3,000 rpm for 10 min, the plasma was transferred and frozen for detection.

### 2.5 UPLC and MS conditions

The chromatographic column was an Acquity BEH C18 column (2.1 mm × 50 mm, 1.7 μm), the mobile phases were 0.1% formic acid and acetonitrile, the flow rate was 0.3 mL/min. The gradient program was as follows: 0.00–0.50 min, with a ratio of 10% acetonitrile; 0.50–1.00 min, with a ratio of 10%–90% of acetonitrile; 1.00–2.00 min, with a ratio of 90% acetonitrile; 2.00–2.10 min, with a ratio of 90%–10% of acetonitrile; 2.10–3.00 min, with a ratio of 10% acetonitrile. The column temperature was set at 45°C and the autosampler was conditioned at 4°C. An injection volume of 2 μL was applied for analysis.

In positive ionization mode, the multiple reaction monitoring (MRM) was used for detection of the four analytes. The MS conditions was summarized in Table 2. All data were acquired in centroid mode by Masslynx V4.1 software (Waters, Milford, MA, United States).

**TABLE 2:** MS parameters of 4 analytes and internal standard.

Analytes	ESI source	RT (min)	Parent ion (m/z)	Daughter ion (m/z)	Dwell (s)	Cone (V)	Collision (V)
ATC	+	1.19	358.1	206.2	0.06	30	15
DEX	+	1.21	201.2	95.1	0.06	30	15
MDZ	+	1.22	326.1	291.1	0.06	10	25
1-OH MDZ	+	1.22	341.9	324.0	0.06	50	20
IS	+	1.44	284.9	153.9	0.06	10	30

### 2.6 Preparation of stock solutions, calibration standards and quality control samples

ATC, DEX, MDZ, 1-OH MDZ, and IS were prepared at a respective concentration of 1.0 mg/mL by dissolving in methanol as the stock solutions, then the stock solutions were diluted separately into application solutions of different concentrations. Calibration curve and QC (quality control) samples were obtained by spiking different volumes of application solutions into different volumes of blank plasma in polypropylene tubes. Different concentrations of plasma mixed standard solutions were prepared to obtain standard curves, including ATC concentrations of 10, 25, 50, 100, 200, 400, 800, 1,600 ng/mL, DEX concentrations of 0.1, 0.25, 0.5, 1, 2.5, 5, 10, 20 ng/mL, and MDZ concentrations of 10, 25, 50, 100, 250, 500, 1,000, 2,000 ng/mL, 1-OH-MDZ concentrations of 10, 25, 50, 100, 250, 500, 1,000, 2,000 ng/mL respectively. At the same time, different concentrations of plasma mixed standard solutions were prepared to obtain QC samples at three concentration levels of low, medium, and high, including ATC concentrations of 25, 400, 1,200 ng/mL, DEX concentrations of 0.25, 5, 15 ng/mL, and MDZ concentrations of 25, 500, 1,500 ng/mL, 1-OH-MDZ concentrations of 25, 500, 1,500 ng/mL, respectively.

### 2.7 Sample preparation

The patient’s plasma sample was processed using acetonitrile precipitation protein method. In a 1.5 mL EP tube, 100 μL of the tested plasma was first added, followed by 10 μL of 1 μg/mL IS application solution, and mixed well. Then 300 μL of acetonitrile was added, vortex mixed for 1 min. Finally, the mixture was centrifuged at 15,000 × *g* for 15 min, then the supernatant was obtained and 2 μL of supernatant was injected to be analyzed by the UPLC-MS/MS system.

### 2.8 Method validation

The bioanalytical methods were validated according to the guidelines for validation of quantitative analytical methods for biological samples in the 2020 edition of the Pharmacopoeia of the People’s Republic of China. The main purpose of validation was to demonstrate the reliability of a specific method for determining the concentration of analytes in a certain biological matrix. The main characteristics of analytical methods include: selectivity, lower limit of quantification (LLOQ), standard curve, accuracy, precision, matrix effects, and stability.

#### 2.8.1 Selectivity

The analysis method should be able to target the endogenous components of the analyte and plasma or other components in the sample. Suitable blank plasma from at least 6 subjects should be used to demonstrate selectivity.

#### 2.8.2 LLOQ

The LLOQ was the minimum concentration of analytes in a sample that could be reliably quantified, with acceptable accuracy and precision. The LLOQ was the lowest point of the standard curve and should be applicable to the expected concentration and experimental purpose.

#### 2.8.3 Standard curve

A standard curve should be obtained by evaluating the response of the instrument to the analyte over a specified concentration range. The calibration standards for each concentration, i.e., plasma mixed standard solution were prepared by adding known concentrations of analytes and internal standards to blank plasma. Each analyte in method validation should have its own standard curve. The calculated concentration of the standard curve should be within 15% of the labeled amount, and the LLOQ should be within 20%.

#### 2.8.4 Accuracy

The accuracy of an analytical method is the degree to which the measured value of the method is close to the indicated concentration of the analyte, expressed as (measured value/true value) * 100%. QC samples should be used to evaluate accuracy. The accuracy should include the LLOQ within the same batch and between different batches, as well as the accuracy of low, medium, and high QC samples.

#### 2.8.5 Precision

The precision of an analytical method describes the degree of closeness to repeated measurements of the analyte, defined as the relative standard deviation (RSD) of the measured value. The results of the analytical batch samples that are similar to the accuracy proof should be used to obtain the LLOQ within the same batch and between different batches, as well as the precision of low, medium, and high QC samples.

#### 2.8.6 Matrix effects

At least 6 batches of blank matrices from different subjects should be used to investigate matrix effects. By calculating the ratio of peak area in the presence of matrix to the corresponding peak area without matrix, the matrix effect of each analyte and internal standard is calculated.

#### 2.8.7 Stability

Stability must be ensured at every step of the analysis method. Stability tests should usually be conducted under the following conditions: room temperature storage, 3 freeze-thaw cycles, long-term freezing storage, and stability of the sample after treatment.

### 2.9 Plasma sample testing

Batch processing method was used to detect plasma samples from patients, with each analysis batch including standard curves, plasma to be tested, and QC samples.

### 2.10 Data analysis

The pharmacokinetic parameters of each analyte were calculated by the statistical moment (noncompartmental analysis) using the DAS 2.0 software package. All data were expressed as mean ± standard deviation (SD).

## 3 Results and discussion

### 3.1 Methodology optimization

Acetonitrile is a commonly used extraction solvent that has good extraction efficiency for most compounds. Lowering the pH value of the extraction solvent can damage the tissue structure of the sample, allowing the target substance to free from the tissue and accelerate the extraction efficiency; At the same time, acetonitrile precipitation can effectively remove plasma proteins, reduce the impact on the chromatographic column and interference in detection.

Vortex oscillation extraction can fully mix the solvent and sample matrix, and the effect of vortex oscillation extraction time (5, 10, 15 min) on extraction efficiency was experimentally investigated. The results showed that the extraction effect significantly increased with the extension of time between 5 and 10 min, and after 10 min, the extraction effect tended to stabilize with the extension of time. Therefore, vortex oscillation for 10 min was selected as the sample extraction time.

The chemical structures and properties of the four analytes differ greatly. In this experiment, UPLC was used and BEH C18 chromatography column was selected to separate the target substance. Adding formic acid to the mobile phase can increase the retention capacity of the chromatographic column and improve separation efficiency. When using acetonitrile 0.1% formic acid solution as the mobile phase, the target substance has a good chromatographic separation effect and a good response value. Further optimization of the gradient elution program resulted in better separation of the target substance, shortened sample analysis time, and improved analysis efficiency.

The mixed standard solution was prepared and subjected to primary full scan mass spectrometry in positive ion mode to obtain the parent ion. The optimal cone hole voltage and capillary voltage were selected through debugging, and then the parent ion was subjected to secondary mass spectrometry analysis to obtain daughter ion characteristic fragments. Two pairs of ion pairs with high response values and low baseline noise in the characteristic fragments were selected as quantitative ions, and the collision energy of daughter ion pairs was optimized to maximize their abundance. Further optimize the mass spectrometry parameters such as Dwell time, cone voltage, and collision voltage to achieve the best ionization efficiency and obtain the optimal mass spectrometry conditions.

### 3.2 Method validation

**FIGURE 2:**
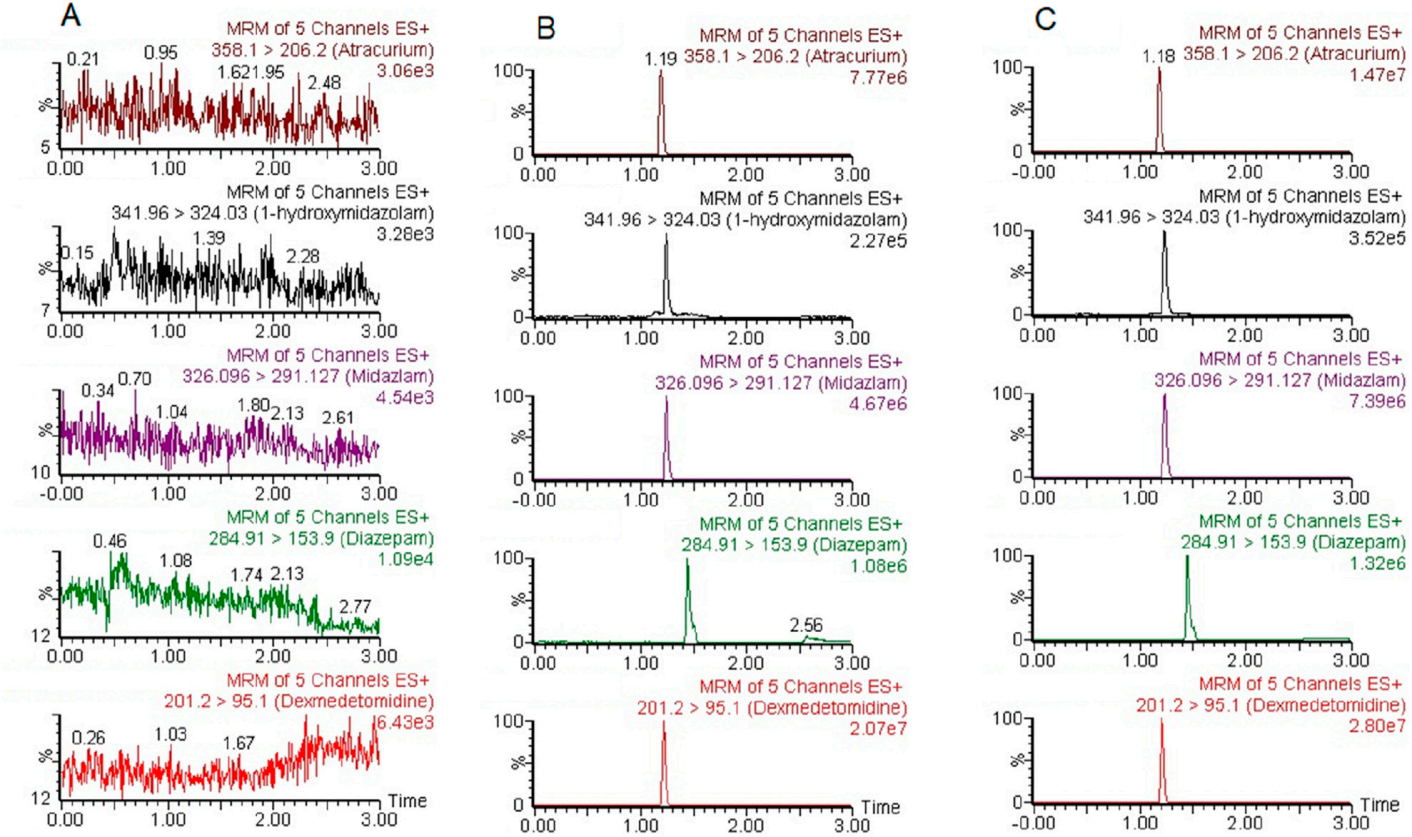
Representative chromatograms of ATC, DEX, MDZ, 1-OH-MDZ, and IS. **(A)** a blank plasma sample; **(B)** a blank plasma sample spiked with ATC, DEX, MDZ, 1-OH-MDZ, and IS; **(C)** a patient plasma sample.

Under the experimental conditions already described, ATC, DEX, MDZ, 1-OH-MDZ, and IS were all significantly separated from endogenous substances. Representative chromatograms of a blank plasma sample (A), a plasma sample spiked with the four analytes and IS (B), and a patient sample (C) were shown in Figure 2. The retention times for ATC, DEX, MDZ, 1-OH-MDZ, and IS were 1.19, 1.21, 1.24, 1.24, and 1.44 min, respectively. The respective total run time for each sample was 3.0 min.

The regression equations, correlation coefficients (R), linear ranges and LLOQs of ATC, DEX, MDZ and 1-OH-MDZ were given in Table 3, which all showed good linear relationships.

**TABLE 3:** Regression equation, linear ranges, R and LLOQ of four analytes.

Analytes	Regression equation	Linear ranges (ng/mL)	R	LLOQ (ng/mL)
ATC	*y* = 0.0409 *x* + 0.7804	10–1,600	0.999 4	10
DEX	*y* = 0.2089 *x* − 0.0346	0.1–20	0.999 1	0.1
MDZ	*y* = 0.0396 *x* − 0.0849	10–2,000	0.999 3	10
1-OH-MDZ	*y* = 0.0026 *x* + 0.0111	10–2,000	0.999 4	10

The intra and inter batch precision and accuracy results for ATC, DEX, MDZ, 1-OH-MDZ were given in Table 4. The precision (RSD%) for all these analytes did not exceed 11%. The accuracy (%) of all analytes ranged from 94.33% to 104.51% at any of the concentrations studied, meeting the validation requirements.

**TABLE 4:** Precision and accuracy of four analytes in patient plasma (n = 6, Mean ± SD).

Analytes	Spiked (ng/mL)	Intra-day		Inter-day		Matrix effect (%)
		RSD (%)	Accuracy (%)	RSD (%)	Accuracy (%)	
ATC	10	6.10	95.48	7.81	101.51	Not evaluated
	25	7.72	104.51	6.88	98.74	96.44 ± 4.72
	400	2.48	102.20	3.70	101.62	103.25 ± 4.50
	1,200	1.37	98.80	2.41	99.21	98.54 ± 3.02
DEX	0.1	9.87	94.33	10.72	97.56	Not evaluated
	0.25	6.44	104.00	7.32	100.11	98.28 ± 4.91
	5	4.56	99.43	3.80	100.89	101.22 ± 3.32
	15	2.50	101.02	2.99	99.45	102.53 ± 3.15
MDZ	10	6.84	103.98	5.44	97.59	Not evaluated
	25	4.95	98.62	4.12	101.36	98.01 ± 5.37
	500	1.96	101.77	2.98	100.92	102.27 ± 3.49
	1,500	3.37	97.76	4.24	102.09	99.56 ± 5.24
1-OH MDZ	10	5.62	97.83	6.18	104.11	Not evaluated
	25	3.69	101.19	3.93	98.72	102.41 ± 4.66
	500	4.11	98.43	4.13	101.53	99.02 ± 4.60
	1,500	2.93	102.37	3.19	99.68	101.37 ± 5.48

The matrix effect results were investigated and shown in Table 4, which were accepted in accordance with the guidelines for the method validation. No patient plasma measurements of each analyte were found to be affected by matrix effects.

All results for the stability under different conditions were summarized in Table 5, and they were within the acceptable criteria of ± 15%, indicating that ATC, DEX, MDZ and 1-OH-MDZ were stable under the conditions described above.

**TABLE 5:** The stability of the four analytes in patient plasma (n = 6, Mean ± SD).

Analytes	Spiked (ng/mL)	Room temperature, 12 h		Autosampler 4°C, 12 h		Three freeze-thaw		−80°C, 4 weeks	
		RSD (%)	Accuracy (%)	RSD (%)	Accuracy (%)	RSD (%)	Accuracy (%)	RSD (%)	Accuracy (%)
ATC	25	6.21	98.76	5.05	99.69	4.90	101.35	4.35	97.70
	400	2.94	98.47	4.66	101.76	4.47	96.73	3.14	102.91
	1,200	1.64	101.31	3.22	99.06	1.91	98.73	3.05	101.22
DEX	0.25	6.69	105.60	3.24	96.73	2.80	106.93	5.28	98.33
	5	3.77	101.57	3.70	97.80	5.31	102.87	6.09	97.53
	15	4.92	96.62	4.08	102.14	3.31	97.87	4.40	102.23
MDZ	25	6.01	98.77	4.52	103.75	5.01	101.03	2.83	96.72
	500	4.33	100.92	4.65	97.31	3.84	104.05	5.10	98.66
	1,500	4.34	102.55	3.19	98.14	2.30	102.58	2.34	97.18
1-OH-MDZ	25	3.07	102.89	5.06	98.75	6.19	101.58	3.05	96.28
	500	3.47	98.37	5.27	101.48	5.68	96.75	5.13	103.06
	1,500	4.03	101.83	3.94	100.26	2.69	99.71	3.66	98.09

### 3.3 Pharmacokinetic study

**FIGURE 3:**
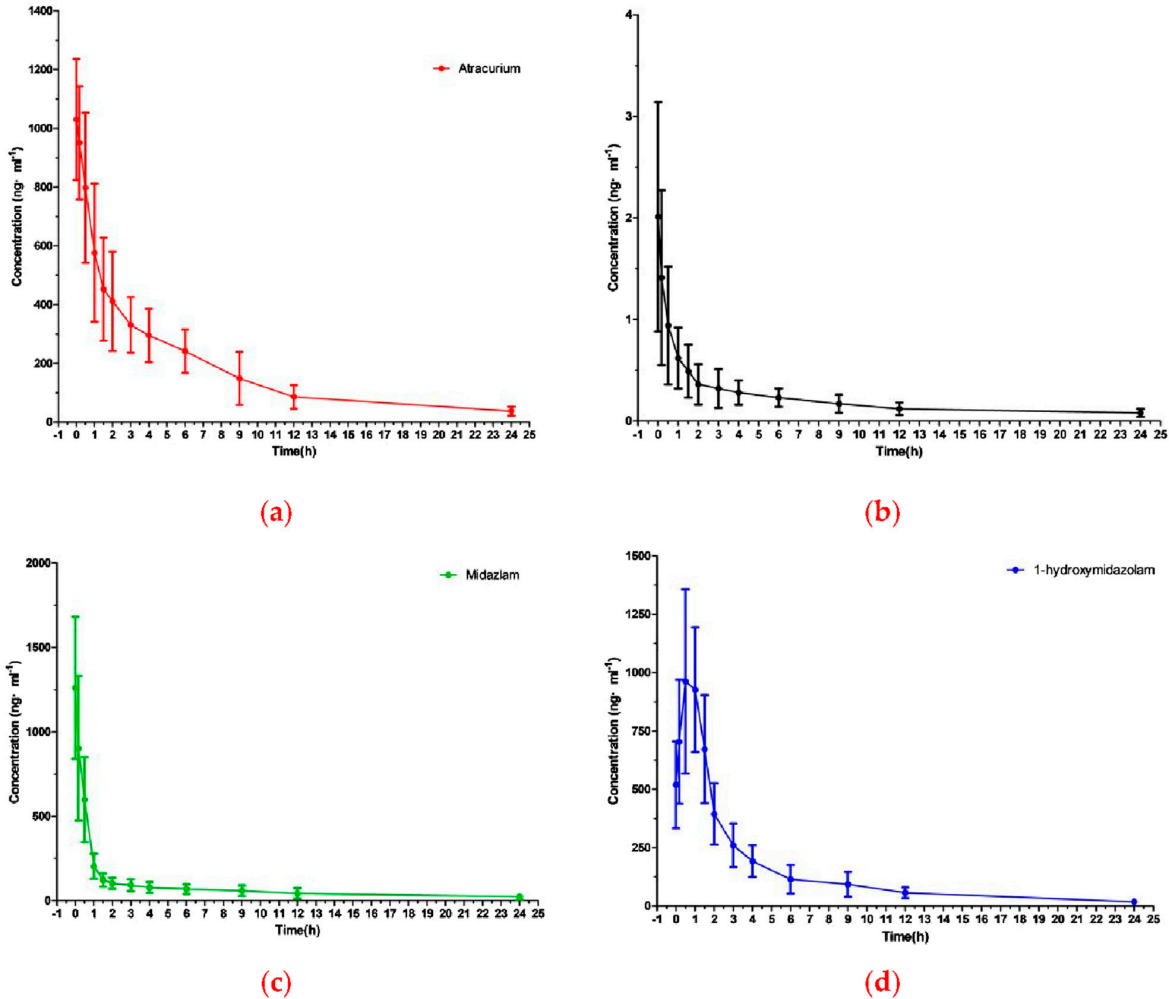
The mean plasma concentration-time curve profile of four analytes: **(A)** ATC; **(B)** DEX; **(C)** MDZ; **(D)** 1-OH-MDZ.

The method described above was successfully applied to a pharmacokinetic study of ATC, DEX, MDZ, and 1-OH-MDZ, which the plasma concentration was determined after simultaneous administration of ATC, DEX, MDZ to patients undergoing acute aortic dissection surgery. The plasma concentration-time profiles of ATC, DEX, MDZ, and 1-OH-MDZ were shown in Figure 3, and the pharmacokinetic parameters of ATC, DEX, MDZ, and 1-OH-MDZ including t_1/2_, MRT_(0−t)_, MRT_(0−∞)_, C_max_, CL, Vd, AUC_(0−t)_ and AUC_(0−∞)_ were listed in Table 6.

**TABLE 6:** Pharmacokinetic parameters of ATC, DEX, MDZ, and 1-OH-MDZ in patients undergoing acute aortic dissection surgery (n = 7, Mean ± SD).

Parameters	ATC	DEX	MDZ	1-OH-MDZ
t_1/2_ (h)	6.05 ± 1.25	9.99 ± 5.15	10.62 ± 5.62	7.02 ± 2.55
MRT_(0-t)_ (h)	5.91 ± 0.54	5.57 ± 1.81	5.73 ± 1.33	4.65 ± 0.77
MRT_(0-∞)_ (h)	7.98 ± 1.06	11.78 ± 6.05	11.82 ± 7.70	6.38 ± 1.78
CL (L/h/kg)	0.025 ± 0.008	0.056 ± 0.028	0.058 ± 0.036	0.030 ± 0.011
Vd (L/kg)	0.22 ± 0.09	0.70 ± 0.33	0.74 ± 0.29	0.32 ± 0.14
C_max_ (ng/mL)	1030.23 ± 205.95	1.99 ± 1.14	1261.03 ± 420.04	1039.83 ± 315.95
AUC_(0-t)_ (ng·h/mL)	4170.09 ± 1370.08	4.98 ± 2.25	1838.06 ± 788.17	3361.75 ± 1127.20
AUC_(0-∞)_ (ng·h/mL)	4452.75 ± 1531.75	6.36 ± 2.37	2295.48 ± 1257.45	3542.24 ± 1138.24

Pharmacokinetic studies showed that after continuous administration of ATC, DEX, and MDZ to patients undergoing surgery for acute aortic dissection, the concentrations of ATC, MDZ, and 1-OH-MDZ were (1030.23 ± 205.95) ng/mL, (1261.03 ± 420.04) ng/mL, and (1039.83 ± 315.95) ng/mL, respectively, while the concentrations of DEX was (1.99 ± 1.14) ng/mL. The first blood collection point for this study was at the end of surgery, at which time ATC, DEX, and MDZ all stopped administration, resulting in a peak time of 0 h 1-OH-MDZ was a metabolite of MDZ, so the concentration of 1-OH-MDZ would increase and then decrease, with a peak time of about 0.69 h, the C_max_ ratio of MDZ to 1-OH-MDZ was 1.21, and the AUC_(0-t)_ ratio was 0.55. This study used intravenous injection of ATC, and there were differences in pharmacokinetic parameters compared to inhalation administration (Beemer et al., 1990).

DEX is an alpha 2 adrenergic receptor agonist and a strong inhibitor of cytochrome P450 enzymes, which can inhibit the hepatic microsomal metabolism of fentanyl drugs (Chang et al., 2019). DEX could inhibit the metabolism of MDZ and increase the exposure of MDZ in beagle dogs, Meanwhile, the plasma exposure of 1-OH-MDZ decreased (Zhou et al., 2020). Therefore, it can be inferred that patients may also experience drug drug interactions after using DEX, ATC, and MDZ simultaneously. Although ATC is predominantly degraded in extrahepatic tissues, but ATC can influence CYP450 (Böhrer et al., 1993). Therefore, in clinical practice, it is important to closely observe potential adverse reactions that may occur.

## 4 Conclusion

In this study, ATC, DEX, MDZ and 1-OH-MDZ were simultaneously determined in patient plasma by the more sensitive UPLC-MS/MS method which requires a simple acetonitrile precipitation step and the analysis time was 3.0 min per sample. After continuous administration of ATC, DEX, and MDZ to patients undergoing surgery for acute aortic dissection, the pharmacokinetics of ATC, DEX, MDZ and 1-OH-MDZ in patients undergoing aortic dissection surgery were studied. In clinical practice, it is important to closely observe potential drug drug interactions that may occur.

## Data Availability

The original contributions presented in the study are included in the article/supplementary material, further inquiries can be directed to the corresponding authors.
